# Novel key cytokines in allergy: IL-17, IL-22* 

**DOI:** 10.5414/ALX01403E

**Published:** 2017-08-04

**Authors:** S. Eyerich, C. Traidl-Hoffmann, H. Behrendt, A. Cavani, C.B. Schmidt-Weber, J. Ring, K. Eyerich

**Affiliations:** 1 ZAUM – Zentrum Allergie & Umwelt, Technische Universität und Helmholtz-Zentrum München, Munich, Germany,; 2 Klinik und Poliklinik für Dermatologie und Allergologie am Biederstein, Technische Universität München, Munich, Germany,; 3 Laboratory of Immunology, Istituto Dermopatico DellImmacolata, Rome, Italy

**Keywords:** immunology, T cell, cytokine, IL-17, IL-22

## Abstract

The biology of the T cell cytokines Interleukin (IL-)17 and IL-22 has been a main focus in the field of clinical immunology in the last decade. This intensive interest in both cytokines has resulted in almost 5,000 scientific publications (www.pubmed.com) dealing with the molecular structure, extra- and intracellular signaling pathways, specific transcription factors and the function of IL-17 and IL-22. This review article highlights the main findings concerning IL-17 and IL-22 in the last years.

*Based on a lecture on the occasion of the 5^th^ German Allergy Congress 2010, Hannover, Germany.

German version published in Allergologie, Vol. 34, No. 7/2011, pp. 344-349

## Cellular source of IL-17 and IL-22 

Although IL-17 and IL-22 have been known since 1993 [1] and 2000 [2], respectively, they did not come under scientific scrutiny until Th17 cells were discovered. In 2006, various researchers described this T-helper cell population as an independent line of T-helper cells [3, 4]. In the past two years it could be shown, however, that IL-17 and IL-22 are also secreted by other leukocytes (Table 1). Leukocytes of the innate immune system like NKT cells, certain populations of NK cells (NK22 cells) [5], as well as follicular T-helper cells [6] have been described as sources of IL-17 and/or IL-22. In 2009, we, and other study groups, could demonstrate the existence of other T-helper cells that secrete IL-22, but not IFN-g, IL-4, or IL-17. These T-helper cells were named Th22 cells in analogy to the established classification [7, 8, 9, 10]. The subject of T cell immunology is becoming more and more complex due to the increasing number of subtypes as well as the phenomenon of plasticity. T cell plasticity is the ability of T cells to change their phenotype or to exhibit characteristics of several phenotypes at the same time. This T cell plasticity is also known for IL-17+ and IL-22+ T cells; more than 10 years ago, CD4+ T cells were described that secrete IL-17 as well as IFN-g (main cytokine of Th1 cells) or IL-4 (that defines Th2 cells) [11]. Th1/IL-17 cells seem to play a key role in psoriasis and allergic contact eczema; Th2/IL-17 cells are characteristic for atopic diseases like atopic eczema [12] or bronchial asthma [13]. So, IL-17 and IL-22 are produced and secreted by a high number of leukocytes. 

## The role of the local microenvironment in the secretion of IL-17 and IL-22 

This observation led to the development of a second research focus, namely the question of whether the cellular source of IL-17 and IL-22 is in fact relevant, or if there are specific processes in the local microenvironment that result in the release of these mediators irrespective of the cell type. Indeed, several molecules that induce the differentiation of Th17 cells from naïve progenitor cells could be identified. It was, for example, shown that certain microbial components (so-called pathogen-associated molecular patterns – PAMPS) of extracellular microorganisms like bacteria [14] and fungi [15] promote Th17 differentiation. Other, non-infectious molecules that promote Th17 differentiation are, for instance, bleomycin [16] and uric acid [17], both activating the inflammasome in immune cells which results in the release of the Th17-induced cytokine IL-1β. In contrast to Th17 differentiation it is, however, widely unknown under which circumstances differentiated Th17 cells actually release IL-17. What could be clearly shown is that the ability of leukocytes to produce IL-17 is linked to the transcription factor RORC (in the murine system: RORgT) [18]. Furthermore, it is probable that strong stimuli are necessary for the secretion of IL-17. Such strong stimuli can, for example, be bacterial superantigens like SEB that stem from *Staphylococcus aureus* [12]. 

SEB does not only induce IL-17 but also the secretion of IL-22 [19]. In addition, the transcription factor aryl hydrocarbon receptor (AHR) was identified to be essential for the secretion of IL-22. Exogenous agonists like dioxins [8, 20] or endogenous agonists like degradation products of the amino acid tryptophan [21] induce the secretion of IL-22. This suggests that IL-22 is an important cytokine at the interface between toxicology and immunology. 

## The functions of IL-17 and IL-22 

IL-17 and IL-22 belong to a new class of cytokines that mainly, or exclusively, affect tissue cells (so-called tissue signaling cytokines) [22]. This pattern is explained by the distribution of specific receptors for IL-17 and IL-22. IL-17 binds as a dimer to the IL-17 receptor A, expressed on almost all epithelial cells of the body and also on immune cells, and/or to the IL-17 receptor C, that is exclusively found on epithelial cells [23]. IL-22 binds, also as a dimer, to a receptor complex composed of the ubiquitously expressed IL-10 receptor B and the IL-22 receptor that is only expressed on epithelial cells [24]. 

**Figure 1 Figure1:**
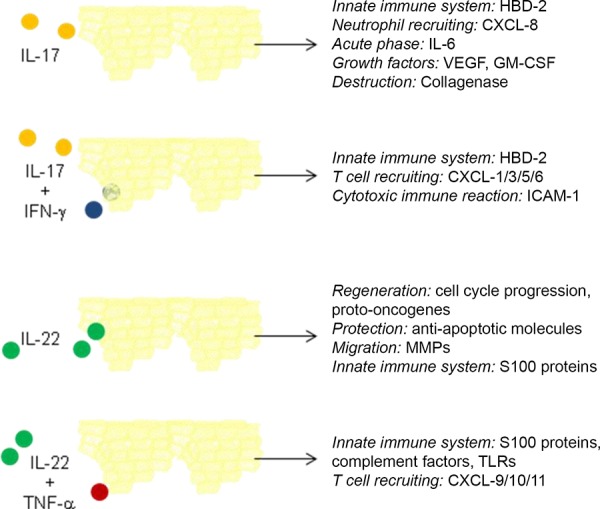
Figure 1. Effects of IL-17 and IL-22 alone and in combination with pro-inflammatory cytokines on epithelial cells. Modified from: Eyerich et al., Trends Immunol. 2010.

The essential function of both cytokines is the induction of an innate immune defense in the peripheral tissue, particularly in the skin and the mucosa. The first hints of this important function were seen when, in 2006, it was discovered that IL-17 and IL-22 syntergistically induce the secretion of the antimicrobial peptide HBD-2 in keratinocytes [25]. Further important information on the primary function of IL-17 and IL-22 was provided by two rare human diseases associated with a loss of both cytokines: autosomal dominant hyper-IgE syndrome [26] and chronic mucocutaneous candidiasis [27]. In both diseases, patients characteristically suffer from chronically relapsing infections of the skin and mucosa with extracellular microorganisms like the fungus *Candida albicans*, while not suffering from systemic infections. The impact of IL-17 and IL-22 on numerous infectious diseases of the skin and mucosa have been described [28].[Fig Figure1]

In addition to infectious diseases, IL-17 and IL-22 also seem to play an important role in autoimmune diseases. It could be shown that Th17 cells are essential in the pathogenesis of experimental autoimmune encephalitis [29] and rheumatoid arthritis [30]. Interestingly, the assumption that these effects of Th17 cells can be traced back to IL-17 and/or IL-22 is not necessarily supported by newer findings. Although for both autoimmune diseases, pathogenic effects of IL-17 could be shown, these were by far weaker than those of Th17 cells [31]. IL-22, on the other hand, showed no pathologic characteristics in several disease models and even had protective effects in experimental myocarditis [32]. Similar observations were made for experimental uveitis [33]. Thus, it seams clear that Th17 cells have to produce further factors that, at least in the murine model, cause several autoimmune diseases; IL-17 is only partially responsible while IL-22 is not responsible for this effect. 

IL-22 has pronounced regenerative and tissue-protective features. This was first described for the liver where IL-22 has protective effects against toxic hepatits [34]. This is due to the induction of anti-apopotitic molecules in hepatocytes [34, 35]. In the lung, IL-22 increases the transepithelial resistance and protects against cellular damage [36]. In the skin, IL-22 induces the proliferation and migration of keratinocytes and inhibits their differentiation; all these effects are essential for wound healing [37]. 

Important information on the function of IL-17 and IL-22 was gained by cell culture models that studied the isolated effect of IL-17 or IL-22 on epithelial cells. Numerous recent studies, however, show that both cytokines interact with other mediators on a functional level. For IL-17, for example, an interaction with the pro-inflammatory IFN-g has been shown. Both cytokines synergistically induce the adhesion molecule ICAM-1 on keratinocytes. This results in an increased binding of T cells to keratinocytes and thus in their unspecific apoptosis [38]. This means that IL-17 enhances an unspecific T cell-mediatiated cytotoxic immune reaction in the skin which seems to be of particular importance in the context of allergic contact dermatitis. IL-22 also interacts with pro-inflammatory cytokines. For example, it enhances the TNF-α-induced secretion of pro-inflammatory chemokines and molecules of the innate immune defense in keratinocytes [39, 40]. 

## The role of IL-17 and IL-22 in allergy 

In allergic inflammation tissue-damaging as well as tissue-repairing processes take place so that IL-17 and IL-22 play an ambivalent role. In this context, it is interesting to note that the Th17-promoting IL-23 also supports the differentiation of Th2 cells [41]. In asthma, IL-17 levels are increased in the lung and this induces IL-6 and IL-11 in fibroblasts [42]. These pro-inflammatory effects of IL-17 seem to be of particular importance in steroid-resistant bronchial asthma [43]. 

The role of IL-22 in allergic inflammation is not yet completely understood. It could be shown that the neutralization of IL-22 inhibits the infiltration of eosinophil but not of neutrophil granulocytes in the lung [44]. In humans, it seems to be clear that IL-22-producing T cells infiltrate into lesions from allergic skin diseases [39] as well as into lung tissue damaged by asthma [unpublished data]. Further investigation has to show whether IL-17 and IL-22 could be an interesting target for to controlling changes of tissue, in particular in chronic allergic reaction. 

## Conclusion 

In general, the effects of IL-17 and IL-22 can be protective – as in the case of extracellular infections – as well as pathogenic – as in psoriasis, allergies, and other inflammatory diseases of the tissue. How both cytokines act in the tissue depends on their concentration as well as on the local microenvironment and on other key cytokines that interact with IL-17 and/or IL-22 in multiple ways. 

**Table 1.** Cells described as sources of IL-17 and/or IL-22.

Cell	Cytokine	Further secreted factors	Surface markers
Th17 [45]	IL-17, IL-22	IL-21, IL-26, TNF-α, (IL-10), CCL20	CD4+ CCR4+ CCR6+CXCR3- CD161+ [46, 47] IL-23R+
			
Th22 [8, 9]	IL-22	TNF-α, (IL-10), FGFs	CD4+ CCR4+ CCR6+CCR10+
			
NKT [48]	IL-17, IL-22	IFN-g	CD3+ CD56+
			
Lymphoid tissue-inducing cell [49]	(IL-17), IL-22	TNF-α, lymphotoxin	CD3- CD56- NKp44- CD117+ CD127+ CD161+
			
RORc+ NKp46+ cells	(IL-17), IL-22		CD3- CD56+ NKp44+ NKp46+ NKG2D+ CD117+ CD127+
			
NK22 [5]	IL-22	TNF-α, lymphotoxin, IL-26	CD3- CD56+ NKp44+ CD117+ CD127+ CD161+
			
CD8+IL-17+ [50]	IL-17		CD3+ CD8+ CD45RO+
			
gd T cell [51]	IL-17		Mouse: CD3+ CD4- CD8- CD27- [52] CD25+ CD122- [53]
			
T follicular helper cell [6]	IL-17	IL-21	CD4+ ICOS+ CXCR5+
			
Monocyte [5^4^, ^55^] Macrophage [55]	IL-17 IL-22		CD11b+ CD68+
			
Neutrophil granulocyte [56]	IL-17		

## References

[1] Rouvier E Luciani MF Mattéi MG Denizot F Golstein P CTLA-8, cloned from an activated T cell, bearing AU-rich messenger RNA instability sequences, and homologous to a herpesvirus saimiri gene. J Immunol. 1993; 150: 5445–5456. 8390535

[2] Dumoutier L Van Roost E Ameye G Michaux L Renauld JC IL-TIF/IL-22: genomic organization and mapping of the human and mouse genes. Genes Immun. 2000; 1: 488–494. 11197690 10.1038/sj.gene.6363716

[3] Weaver CT Harrington LE Mangan PR Gavrieli M Murphy KM Th17: an effector CD4 T cell lineage with regulatory T cell ties. Immunity. 2006; 24: 677–688. 16782025 10.1016/j.immuni.2006.06.002

[4] Schmidt-Weber CB Akdis M Akdis CA TH17 cells in the big picture of immunology. J Allergy Clin Immunol. 2007; 120: 247–254. 17666214 10.1016/j.jaci.2007.06.039

[5] Norian LA Rodriguez PC O’Mara LA Zabaleta J Ochoa AC Cella M Allen PM Tumor-infiltrating regulatory dendritic cells inhibit CD8+ T cell function via L-arginine metabolism. Cancer Res. 2009; 69: 3086–3094. 19293186 10.1158/0008-5472.CAN-08-2826 PMC2848068

[6] Wu HY Quintana FJ Weiner HL Nasal anti-CD3 antibody ameliorates lupus by inducing an IL-10-secreting CD4+ CD25- LAP+ regulatory T cell and is associated with down-regulation of IL-17+ CD4+ ICOS+ CXCR5+ follicular helper T cells. J Immunol. 2008; 181: 6038–6050. 18941193 10.4049/jimmunol.181.9.6038 PMC2753458

[7] Duhen T Geiger R Jarrossay D Lanzavecchia A Sallusto F Production of interleukin 22 but not interleukin 17 by a subset of human skin-homing memory T cells. Nat Immunol. 2009; 10: 857–863. 19578369 10.1038/ni.1767

[8] Trifari S Kaplan CD Tran EH Crellin NK Spits H Identification of a human helper T cell population that has abundant production of interleukin 22 and is distinct from T(H)-17, T(H)1 and T(H)2 cells. Nat Immunol. 2009; 10: 864–871. 19578368 10.1038/ni.1770

[9] Eyerich S Eyerich K Pennino D Carbone T Nasorri F Pallotta S Cianfarani F Odorisio T Traidl-Hoffmann C Behrendt H Durham SR Schmidt-Weber CB Cavani A Th22 cells represent a distinct human T cell subset involved in epidermal immunity and remodeling. J Clin Invest. 2009; 119: 3573–3585. 19920355 10.1172/JCI40202 PMC2786807

[10] Nograles KE Zaba LC Guttman-Yassky E Fuentes-Duculan J Suárez-Fariñas M Cardinale I Khatcherian A Gonzalez J Pierson KC White TR Pensabene C Coats I Novitskaya I Lowes MA Krueger JG Th17 cytokines interleukin (IL)-17 and IL-22 modulate distinct inflammatory and keratinocyte-response pathways. Br J Dermatol. 2008; 159: 1092–1102. 18684158 10.1111/j.1365-2133.2008.08769.x PMC2724264

[11] Albanesi C Cavani A Girolomoni G IL-17 is produced by nickel-specific T lymphocytes and regulates ICAM-1 expression and chemokine production in human keratinocytes: synergistic or antagonist effects with IFN-gamma and TNF-α. J Immunol. 1999; 162: 494–502. 9886425

[12] Eyerich K Pennino D Scarponi C Foerster S Nasorri F Behrendt H Ring J Traidl-Hoffmann C Albanesi C Cavani A IL-17 in atopic eczema: linking allergen-specific adaptive and microbial-triggered innate immune response. J Allergy Clin Immunol. 2009; 123: 59–66.. 19056110 10.1016/j.jaci.2008.10.031

[13] Wang YH Voo KS Liu B Chen CY Uygungil B Spoede W Bernstein JA Huston DP Liu YJ A novel subset of CD4(+) T(H)2 memory/effector cells that produce inflammatory IL-17 cytokine and promote the exacerbation of chronic allergic asthma. J Exp Med. 2010; 207: 2479–2491. 20921287 10.1084/jem.20101376 PMC2964570

[14] Khader SA Bell GK Pearl JE Fountain JJ Rangel-Moreno J Cilley GE Shen F Eaton SM Gaffen SL Swain SL Locksley RM Haynes L Randall TD Cooper AM IL-23 and IL-17 in the establishment of protective pulmonary CD4+ T cell responses after vaccination and during Mycobacterium tuberculosis challenge. Nat Immunol. 2007; 8: 369–377. 17351619 10.1038/ni1449

[15] Huang W Na L Fidel PL Schwarzenberger P Requirement of interleukin-17A for systemic anti-Candida albicans host defense in mice. J Infect Dis. 2004; 190: 624–631. 15243941 10.1086/422329

[16] Braun RK Ferrick C Neubauer P Sjoding M Sterner-Kock A Kock M Putney L Ferrick DA Hyde DM Love RB IL-17 producing gammadelta T cells are required for a controlled inflammatory response after bleomycin-induced lung injury. Inflammation. 2008; 31: 167–179. 18338242 10.1007/s10753-008-9062-6

[17] Gasse P Riteau N Charron S Girre S Fick L Pétrilli V Tschopp J Lagente V Quesniaux VF Ryffel B Couillin I Uric acid is a danger signal activating NALP3 inflammasome in lung injury inflammation and fibrosis. Am J Respir Crit Care Med. 2009; 179: 903–913. 19218193 10.1164/rccm.200808-1274OC

[18] Manel N Unutmaz D Littman DR The differentiation of human T(H)-17 cells requires transforming growth factor-beta and induction of the nuclear receptor RORgammat. Nat Immunol. 2008; 9: 641–649. 18454151 10.1038/ni.1610 PMC2597394

[19] Niebuhr M Scharonow H Gathmann M Mamerow D Werfel T Staphylococcal exotoxins are strong inducers of IL-22: A potential role in atopic dermatitis. J Allergy Clin Immunol. 2010; 126: 1176–83.. 20864149 10.1016/j.jaci.2010.07.041

[20] Veldhoen M Hirota K Westendorf AM Buer J Dumoutier L Renauld JC Stockinger B The aryl hydrocarbon receptor links TH17-cell-mediated autoimmunity to environmental toxins. Nature. 2008; 453: 106–109. 18362914 10.1038/nature06881

[21] Esser C Rannug A Stockinger B The aryl hydrocarbon receptor in immunity. Trends Immunol. 2009; 30: 447–454. 19699679 10.1016/j.it.2009.06.005

[22] Eyerich S Eyerich K Cavani A Schmidt-Weber C IL-17 and IL-22: siblings, not twins. Trends Immunol. 2010; 31: 354–361. 20691634 10.1016/j.it.2010.06.004

[23] Moseley TA Haudenschild DR Rose L Reddi AH Interleukin-17 family and IL-17 receptors. Cytokine Growth Factor Rev. 2003; 14: 155–174. 12651226 10.1016/s1359-6101(03)00002-9

[24] Wolk K Kunz S Witte E Friedrich M Asadullah K Sabat R IL-22 increases the innate immunity of tissues. Immunity. 2004; 21: 241–254. 15308104 10.1016/j.immuni.2004.07.007

[25] Liang SC Tan XY Luxenberg DP Karim R Dunussi-Joannopoulos K Collins M Fouser LA Interleukin (IL)-22 and IL-17 are coexpressed by Th17 cells and cooperatively enhance expression of antimicrobial peptides. J Exp Med. 2006; 203: 2271–2279. 16982811 10.1084/jem.20061308 PMC2118116

[26] Milner JD Brenchley JM Laurence A Freeman AF Hill BJ Elias KM Kanno Y Spalding C Elloumi HZ Paulson ML Davis J Hsu A Asher AI O’Shea J Holland SM Paul WE Douek DC Impaired T(H)17 cell differentiation in subjects with autosomal dominant hyper-IgE syndrome. Nature. 2008; 452: 773–776. 18337720 10.1038/nature06764 PMC2864108

[27] Eyerich K Foerster S Rombold S Seidl HP Behrendt H Hofmann H Ring J Traidl-Hoffmann C Patients with chronic mucocutaneous candidiasis exhibit reduced production of Th17-associated cytokines IL-17 and IL-22. J Invest Dermatol. 2008; 128: 2640–2645. 18615114 10.1038/jid.2008.139

[28] Miossec P IL-17 and Th17 cells in human inflammatory diseases. Microbes Infect. 2009; 11: 625–630. 19371791 10.1016/j.micinf.2009.04.003

[29] Reboldi A Coisne C Baumjohann D Benvenuto F Bottinelli D Lira S Uccelli A Lanzavecchia A Engelhardt B Sallusto F C-C chemokine receptor 6-regulated entry of TH-17 cells into the CNS through the choroid plexus is required for the initiation of EAE. Nat Immunol. 2009; 10: 514–523. 19305396 10.1038/ni.1716

[30] Murphy CA Langrish CL Chen Y Blumenschein W McClanahan T Kastelein RA Sedgwick JD Cua DJ Divergent pro- and antiinflammatory roles for IL-23 and IL-12 in joint autoimmune inflammation. J Exp Med. 2003; 198: 1951–1957. 14662908 10.1084/jem.20030896 PMC2194162

[31] Haak S Croxford AL Kreymborg K Heppner FL Pouly S Becher B Waisman A IL-17A and IL-17F do not contribute vitally to autoimmune neuro-inflammation in mice. J Clin Invest. 2009; 119: 61–69. 19075395 10.1172/JCI35997 PMC2613466

[32] Chang H Hanawa H Liu H Yoshida T Hayashi M Watanabe R Abe S Toba K Yoshida K Elnaggar R Minagawa S Okura Y Kato K Kodama M Maruyama H Miyazaki J Aizawa Y Hydrodynamic-based delivery of an interleukin-22-Ig fusion gene ameliorates experimental autoimmune myocarditis in rats. J Immunol. 2006; 177: 3635–3643. 16951323 10.4049/jimmunol.177.6.3635

[33] Peng Y Han G Shao H Wang Y Kaplan HJ Sun D Characterization of IL-17+ interphotoreceptor retinoid-binding protein-specific T cells in experimental autoimmune uveitis. Invest Ophthalmol Vis Sci. 2007; 48: 4153–4161. 17724201 10.1167/iovs.07-0251 PMC2567912

[34] Radaeva S Sun R Pan HN Hong F Gao B Interleukin 22 (IL-22) plays a protective role in T cell-mediated murine hepatitis: IL-22 is a survival factor for hepatocytes via STAT3 activation. Hepatology. 2004; 39: 1332–1342. 15122762 10.1002/hep.20184

[35] Pan H Hong F Radaeva S Gao B Hydrodynamic gene delivery of interleukin-22 protects the mouse liver from concanavalin A-, carbon tetrachloride-, and Fas ligand-induced injury via activation of STAT3. Cell Mol Immunol. 2004; 1: 43–49. 16212920

[36] Aujla SJ Chan YR Zheng M Fei M Askew DJ Pociask DA Reinhart TA McAllister F Edeal J Gaus K Husain S Kreindler JL Dubin PJ Pilewski JM Myerburg MM Mason CA Iwakura Y Kolls JK IL-22 mediates mucosal host defense against Gram-negative bacterial pneumonia. Nat Med. 2008; 14: 275–281. 18264110 10.1038/nm1710 PMC2901867

[37] Li W Danilenko DM Bunting S Ganesan R Sa S Ferrando R Wu TD Kolumam GA Ouyang W Kirchhofer D The serine protease marapsin is expressed in stratified squamous epithelia and is up-regulated in the hyperproliferative epidermis of psoriasis and regenerating wounds. J Biol Chem. 2009; 284: 218–228. 18948266 10.1074/jbc.M806267200

[38] Pennino D Eyerich K Scarponi C Carbone T Eyerich S Nasorri F Garcovich S Traidl-Hoffmann C Albanesi C Cavani A IL-17 amplifies human contact hypersensitivity by licensing hapten nonspecific Th1 cells to kill autologous keratinocytes. J Immunol. 2010; 184: 4880–4888. 20357258 10.4049/jimmunol.0901767

[39] Eyerich S Eyerich K Pennino D Carbone T Nasorri F Pallotta S Cianfarani F Odorisio T Traidl-Hoffmann C Behrendt H Durham SR Schmidt-Weber CB Cavani A Th22 cells represent a distinct human T cell subset involved in epidermal immunity and remodeling. J Clin Invest. 2009; 119: 3573–3585. 19920355 10.1172/JCI40202 PMC2786807

[40] Wolk K Haugen HS Xu W Witte E Waggie K Anderson M Vom Baur E Witte K Warszawska K Philipp S Johnson-Leger C Volk HD Sterry W Sabat R IL-22 and IL-20 are key mediators of the epidermal alterations in psoriasis while IL-17 and IFN-gamma are not. J Mol Med (Berl). 2009; 87: 523–536. 19330474 10.1007/s00109-009-0457-0

[41] Peng J Yang XO Chang SH Yang J Dong C IL-23 signaling enhances Th2 polarization and regulates allergic airway inflammation. Cell Res. 2010; 20: 62–71. 19935773 10.1038/cr.2009.128 PMC2801763

[42] Molet S Hamid Q Davoine F Nutku E Taha R Pagé N Olivenstein R Elias J Chakir J IL-17 is increased in asthmatic airways and induces human bronchial fibroblasts to produce cytokines. J Allergy Clin Immunol. 2001; 108: 430–438. 11544464 10.1067/mai.2001.117929

[43] McKinley L Alcorn JF Peterson A Dupont RB Kapadia S Logar A Henry A Irvin CG Piganelli JD Ray A Kolls JK TH17 cells mediate steroid-resistant airway inflammation and airway hyperresponsiveness in mice. J Immunol. 2008; 181: 4089–4097. 18768865 10.4049/jimmunol.181.6.4089 PMC3638757

[44] Schnyder B Lima C Schnyder-Candrian S Interleukin-22 is a negative regulator of the allergic response. Cytokine. 2010; 50: 220–227. 20194033 10.1016/j.cyto.2010.02.003

[45] Acosta-Rodriguez EV Rivino L Geginat J Jarrossay D Gattorno M Lanzavecchia A Sallusto F Napolitani G Surface phenotype and antigenic specificity of human interleukin 17-producing T helper memory cells. Nat Immunol. 2007; 8: 639–646. 17486092 10.1038/ni1467

[46] Cosmi L De Palma R Santarlasci V Maggi L Capone M Frosali F Rodolico G Querci V Abbate G Angeli R Berrino L Fambrini M Caproni M Tonelli F Lazzeri E Parronchi P Liotta F Maggi E Romagnani S Annunziato F Human interleukin 17-producing cells originate from a CD161+CD4+ T cell precursor. J Exp Med. 2008; 205: 1903–1916. 18663128 10.1084/jem.20080397 PMC2525581

[47] Kleinschek MA Boniface K Sadekova S Grein J Murphy EE Turner SP Raskin L Desai B Faubion WA de Waal Malefyt R Pierce RH McClanahan T Kastelein RA Circulating and gut-resident human Th17 cells express CD161 and promote intestinal inflammation. J Exp Med. 2009; 206: 525–534. 19273624 10.1084/jem.20081712 PMC2699125

[48] Rachitskaya AV Hansen AM Horai R Li Z Villasmil R Luger D Nussenblatt RB Caspi RR Cutting edge: NKT cells constitutively express IL-23 receptor and RORgammat and rapidly produce IL-17 upon receptor ligation in an IL-6-independent fashion. J Immunol. 2008; 180: 5167–5171. 18390697 10.4049/jimmunol.180.8.5167 PMC2442579

[49] Cupedo T Crellin NK Papazian N Rombouts EJ Weijer K Grogan JL Fibbe WE Cornelissen JJ Spits H Human fetal lymphoid tissue-inducer cells are interleukin 17-producing precursors to RORC+ CD127+ natural killer-like cells. Nat Immunol. 2009; 10: 66–74. 19029905 10.1038/ni.1668

[50] Shin HC Benbernou N Esnault S Guenounou M Expression of IL-17 in human memory CD45RO+ T lymphocytes and its regulation by protein kinase A pathway. Cytokine. 1999; 11: 257–266. 10328864 10.1006/cyto.1998.0433

[51] Peng MY Wang ZH Yao CY Jiang LN Jin QL Wang J Li BQ Interleukin 17-producing gamma delta T cells increased in patients with active pulmonary tuberculosis. Cell Mol Immunol. 2008; 5: 203–208. 18582402 10.1038/cmi.2008.25 PMC4651291

[52] Ribot JC deBarros A Pang DJ Neves JF Peperzak V Roberts SJ Girardi M Borst J Hayday AC Pennington DJ Silva-Santos B CD27 is a thymic determinant of the balance between interferon-gamma- and interleukin 17-producing gammadelta T cell subsets. Nat Immunol. 2009; 10: 427–436. 19270712 10.1038/ni.1717 PMC4167721

[53] Shibata K Yamada H Nakamura R Sun X Itsumi M Yoshikai Y Identification of CD25+ gamma delta T cells as fetal thymus-derived naturally occurring IL-17 producers. J Immunol. 2008; 181: 5940–5947. 18941182 10.4049/jimmunol.181.9.5940

[54] Zheng Y Danilenko DM Valdez P Kasman I Eastham-Anderson J Wu J Ouyang W Interleukin-22, a T(H)17 cytokine, mediates IL-23-induced dermal inflammation and acanthosis. Nature. 2007; 445: 648–651. 17187052 10.1038/nature05505

[55] Fujino S Andoh A Bamba S Ogawa A Hata K Araki Y Bamba T Fujiyama Y Increased expression of interleukin 17 in inflammatory bowel disease. Gut. 2003; 52: 65–70. 12477762 10.1136/gut.52.1.65 PMC1773503

[56] Ferretti S Bonneau O Dubois GR Jones CE Trifilieff A IL-17, produced by lymphocytes and neutrophils, is necessary for lipopolysaccharide-induced airway neutrophilia: IL-15 as a possible trigger. J Immunol. 2003; 170: 2106–2112. 12574382 10.4049/jimmunol.170.4.2106

